# Skeletal Muscle Insulin Sensitivity Show Circadian Rhythmicity Which Is Independent of Exercise Training Status

**DOI:** 10.3389/fphys.2018.01198

**Published:** 2018-08-28

**Authors:** Astrid L. Basse, Emilie Dalbram, Louise Larsson, Zach Gerhart-Hines, Juleen R. Zierath, Jonas T. Treebak

**Affiliations:** ^1^Section of Integrative Physiology, Novo Nordisk Foundation Center for Basic Metabolic Research, University of Copenhagen, Copenhagen, Denmark; ^2^Section for Metabolic Receptology, Novo Nordisk Foundation Center for Basic Metabolic Research, University of Copenhagen, Copenhagen, Denmark; ^3^Department of Biomedical Sciences, University of Copenhagen, Copenhagen, Denmark; ^4^Section of Integrative Physiology, Department of Molecular Medicine and Surgery, Karolinska Institutet, Stockholm, Sweden

**Keywords:** circadian rhythm, insulin sensitivity, skeletal muscle, exercise training, glucose uptake, insulin tolerance, glucose disposal, adipose tissue

## Abstract

Circadian rhythms can be perturbed by shift work, travel across time zones, many occupational tasks, or genetic mutations. Perturbed circadian rhythms are associated with the increasing problem of obesity, metabolic dysfunction, and insulin resistance. We hypothesized that insulin sensitivity in skeletal muscle follows a circadian pattern and that this pattern is important for overall metabolic function. This hypothesis was verified using mice as a model system. We observed circadian rhythmicity in whole body insulin tolerance, as well as in signaling pathways regulating insulin- and exercise-induced glucose uptake in skeletal muscle, including AKT, 5′-adenosine monophosphate-activated protein kinase (AMPK) and TBC1 domain family member 4 (TBC1D4) phosphorylation. Basal and insulin-stimulated glucose uptake in skeletal muscle and adipose tissues *in vivo* also differed between day- and nighttime. However, the rhythmicity of glucose uptake differed from the rhythm of whole-body insulin tolerance. These results indicate that neither skeletal muscle nor adipose tissue play a major role for the circadian rhythmicity in whole-body insulin tolerance. To study the circadian pattern of insulin sensitivity directly in skeletal muscle, we determined glucose uptake under basal and submaximal insulin-stimulated conditions *ex vivo* every sixth hour. Both insulin sensitivity and signaling of isolated skeletal muscle peaked during the dark period. We next examined the effect of exercise training on the circadian rhythmicity of insulin sensitivity. As expected, voluntary exercise training enhanced glucose uptake in skeletal muscle. Nevertheless, exercise training did not affect the circadian rhythmicity of skeletal muscle insulin sensitivity. Taken together, our results provide evidence that skeletal muscle insulin sensitivity exhibits circadian rhythmicity.

## Introduction

To anticipate changes in physical activity and nutritional requirements, whole-body homeostasis is regulated by intrinsic cellular clocks that maintain daily rhythms of about 24 h (Eckel-Mahan and Sassone-Corsi, 2013; Eckel-Mahan et al., 2013). Perturbation of these rhythms due to shift-work, travel across time zones, many occupational tasks, or genetic mutations is associated with metabolic dysfunction and decreased metabolic health (Karlsson et al., 2003; Eckel-Mahan et al., 2013). The main zeitgeber that regulates circadian rhythms is light, but other external cues such as food intake and physical activity also entrain the cellular clocks. At the molecular level, cell-autonomous circadian rhythms are generated by a transcriptionally auto-regulatory feedback loop. The positive arm in this feedback loop is composed of the transcriptional activators Circadian locomotor output cycles kaput (CLOCK) and Brain and muscle ARNTL-like protein 1 (BMAL1) (Sahar and Sassone-Corsi, 2012; Panda, 2016).

Intrinsic molecular clocks play an important role for glucose metabolism and insulin sensitivity. Whole body knockout of *Clock* or *Bmal1* renders mice prone to obesity and hyperglycemia (Turek et al., 2005; Shi et al., 2013). Furthermore, CLOCK and BMAL1 transgenic mice have decreased insulin tolerance and impaired gluconeogenesis (Rudic et al., 2004). The circadian clock regulates glucose metabolism through tissue-specific mechanisms. Liver-specific knockout of *Bmal1* induces hypoglycemia and decreases glucose and insulin tolerance during the fasting phase in inactive mice (Lamia et al., 2008). Conversely, skeletal muscle-specific ablation of *Bmal1* does not affect glycemia or insulin tolerance (Dyar et al., 2014), but impairs insulin-stimulated skeletal muscle glucose uptake and whole-body glucose tolerance (Dyar et al., 2014; Harfmann et al., 2016). In addition, pancreatic-specific knockout of *Bmal1* impairs glucose tolerance and glucose-stimulated insulin release, while retaining insulin tolerance (Sadacca et al., 2011). These findings emphasize the importance of the clock machinery for tissue-specific regulation of glucose handling, but the circadian rhythmicity of insulin tolerance was not investigated in these studies.

Blood glucose levels and glucose metabolism follow a diurnal rhythm. In mouse models, during the active, dark phase, metabolic intermediates such as glucose are mainly used for lipid synthesis and storage, whereas in the inactive, light phase, glucose production from the liver compensates for the caloric restriction. This shift in metabolic fuel utilization is reflected in increased fasting blood glucose levels and decreased insulin sensitivity in mice during the inactive time of the day (Shi et al., 2013). In healthy humans, glucose tolerance is diminished in the evening compared to the morning. This shift in metabolism is ascribed to both decreased insulin sensitivity of peripheral tissues and reduced responsiveness of insulin producing β-cell in the evening (Lee et al., 1992; Saad et al., 2012). However, it is not clear to what extent the diurnal pattern in whole body insulin sensitivity is reflected at the tissue-specific level.

The phase of the circadian rhythm, both centrally in the brain and peripherally in metabolic organs, shifts in response to exercise (Wolff and Esser, 2012; Yasumoto et al., 2015). Evidence is emerging that exercise training at specific times of the day can correct a “misalignment” of the clock (Mrosovsky and Salmon, 1987; Schroeder et al., 2012). However, the effect of exercise training on circadian rhythm of insulin sensitivity is unknown. We hypothesized that exercise training can potentiate entrainment of the peripheral clock in skeletal muscle and regulate insulin sensitivity in a circadian optimal manner.

In this study, we determined whether rhythmicity of whole-body insulin tolerance was reflected by the putative circadian rhythmicity of skeletal muscle insulin sensitivity. We provide evidence that insulin- and exercise-induced signaling pathways controlling skeletal muscle glucose uptake were augmented during the dark phase in mice. Moreover, whole-body insulin tolerance was high when skeletal muscle insulin sensitivity to glucose uptake was low, and exercise training did not affect the circadian rhythmicity of skeletal muscle insulin sensitivity. Collectively, our data reveal circadian rhythmicity of insulin sensitivity and signaling in skeletal muscle.

## Materials and Methods

### Mouse Studies

Animal experiments complied with the European directive 2010/63/EU of the European Parliament and were approved by the Danish Animal Experiments Inspectorate (2015-15-0201-00792). C57BL/6JBomTac male mice were obtained from Taconic (Denmark). Mice were group-housed, but for exercise training experiments, mice were single-housed. Mice were kept in an enriched environment with a 12 h light:12 h dark cycle with *ad libitum* feed (#1310, Altromin) and water in a temperature-controlled (22°C ± 1°C) room.

### Measurement of Blood Parameters

Blood samples were taken from the tail vein. Blood glucose and lactate levels were measured directly with Contour Next (Bayer) and Lactate Plus (Nova Biochemical) strips, respectively. Insulin and leptin levels were measured by a MULTI-SPOT Assay System (#K15124C, Meso Scale Discovery) according to the instructions from the manufacturer. This assay detects leptin and insulin in a multiplexed sandwich immunoassay. The sample and a solution containing labeled detection antibodies were added to a plate that was pre-coated with leptin and insulin capture antibodies on spatially distinct spots. Reading buffer was added and intensity of emitted light was measured on a QuickPlex SQ 120 (Meso Scale Discovery) to obtain a quantitative measure of leptin and insulin present in the sample.

### Insulin Tolerance Test (ITT)

Mice were fasted for 2 h before an intraperitoneal injection of insulin (0.75 U/Kg bodyweight; Actrapid, Novo Nordisk, Denmark). Blood glucose was measured before and 15, 30, 45, 60, and 120 min after the insulin administration. Separate groups of mice were used for each of the four time-of-the-day ITT experiments performed. The ITTs were performed at least 24 h after the last training bout.

### Western Blot Analyses

The procedures used for protein extraction from skeletal muscle and Western blot analyses have previously been described (Brandauer et al., 2015). Protein concentration was determined by the bicinchoninic acid (BCA) assay (#23225, Pierce). Samples were loaded according to protein concentration and resolved by sodium dodecyl sulfate-polyacrylamide gel electrophoresis (SDS-PAGE). Ponceau S staining was performed to confirm that equal amounts of protein were transferred to each membrane. Western blot analysis was performed by loading samples representing all experimental conditions on each gel. All gels included one or two internal control samples used for normalization purposes in order to allow comparison of samples resolved on separate gels. All gels were cut in smaller pieces corresponding to the specific protein target, and gel pieces were transferred to the same membrane and developed together. Protein abundance was detected using antibodies listed in **Table 1**.

**Table 1:** Antibodies used for Western blot analyses.

Target	Company	Catalog number
ACC-pS79	Cell Signaling	3661
AKT-total	Cell Signaling	9272
AKT-pT308	Cell Signaling	9275L
AKT-pS473	Cell Signaling	9271L
AMPKα2-total	Kindly provided by Prof. D. Grahame Hardie (University of Dundee, United Kingdom)	
AMPKα2-pT172	Cell Signaling	2531s
GLUT4	Thermo Fisher Scientific	PA1-1065
GSK3β-pS 9	Cell Signaling	9336
Hexokinase II	Cell Signaling	2867
Hexokinase II (**Figure 4** Soleus)	Santa Cruz Biotechnology	130358
P70 S6 Kinase-pT389	Cell Signaling	9205
TBC1D1-total	Kindly provided by Prof. Carol Mackintosh (University of Dundee, United Kingdom)	
TBC1D1-pS231	Millipore	07-2268
TBC1D1-pT590	Cell Signaling	6927
TBC1D4-total	Millipore	07-714
TBC1D4-pT642	Cell Signaling	8881
TBC1D4-pS711	Capra Science	Custom made


### *In vivo* Glucose Uptake

Mice were fasted for 2 h before they were anesthetized with 75 mg pentobarbital/kg body weight and glucose uptake in skeletal muscle and adipose tissue was assessed in response to a single retro-orbital injection of ^3^H-2-deoxyglucose (^3^H-2-DG) [12.32 MBq/kg bodyweight (PerkinElmer, #NET549A005MC)] combined with insulin (0.75 U/kg bodyweight; Actrapid, Novo Nordisk, Denmark) or saline dissolved in Gelofusine (B. Braun, Denmark). Total blood glucose levels were determined by spectrophotometric measurements. A reaction mix consisting of 200 mM Tris-HCl, 500 mM MgCl_2_, 5.2 mM adenosine triphosphate (ATP), 2.8 mM nicotinamide adenine dinucleotide phosphate (NADP) (#14469130, Roche) and 148 μg of a hexokinase and glucose-6-phosphate dehydrogenase mixture (#10737275001, Roche) with a pH of 7.4 was added to each sample. The samples were incubated at room temperature for 15 min and absorbance was measured (Hidex Sense, Hidex). Glucose uptake was determined 15 min after the injection as described (Treebak et al., 2010) by measuring the accumulation of ^3^H-2-DG in specific tissues. Radioactivity was determined in lysates by liquid scintillation counting (Hidex 300SL, Hidex) and related to tissue weight input.

### Glucose Uptake *ex vivo* in Incubated Skeletal Muscles

Extensor digitorum longus (EDL) and soleus muscles were dissected from sedentary mice and mice that had free access to a running wheel for 4 weeks. Mice were anesthetized by injections with Avertin [2,2,2-Tribromoethanol (Sigma-Aldrich #T48402) and 2-Methyl-2-butanol 99% (Sigma-Aldrich #152463)]. Muscles were incubated at 30°C in oxygenated (95% O_2_ and 5% CO_2_) pre-buffer [Krebs-Ringer buffer (KRB); 117 mM NaCl, 4.7 mM KCl, 2.5 mM CaCl_2_, 1.2 mM KH_2_PO_4_, 1.2 mM MgSO_4_, 24.6 mM NaHCO_3_ supplemented with 0.1% bovine serum albumin, 8 mM mannitol, and 2 mM pyruvate], in a myograph system (820MS, DMT, Denmark). After 10 min pre-incubation, the pre-buffer was changed to a buffer containing 200 μU/mL insulin (Actrapid, Novo Nordisk, Denmark) or vehicle and a tension of 5 mN per muscle was applied. After 20 min, the buffer was changed to a buffer containing: 1 mM of 2-DG and ^3^H-2-DG (0.0185 MBq/mL, PerkinElmer, #NET549A005MC) together with 7 mM of mannitol and ^14^C-mannitol (0.0167 MBq/mL, PerkinElmer, #NEC314250UC). Muscles were incubated in this buffer for 10 min. After the end of the protocol, muscles were harvested, snap frozen in liquid nitrogen and kept at -80°C.

Protein extraction from muscles was performed as described (Brandauer et al., 2015). Muscle lysate (250 μL) or tracer media (25 μL) was added to 3 mL Ultima Gold^TM^ scintillation liquid (PerkinElmer, #6013326). The vials were counted in a scintillation counter (Hidex 300SL, Hidex) using the MicroWin 2000 software for counting both ^3^H and ^14^C isotopes. Disintegrations per min (DPM) of ^14^C-labeled mannitol in the media and skeletal muscle was used to calculate the extracellular space, by dividing the true activity of the skeletal muscle with the concentration of the media. The fraction of ^3^H-labeled 2-DG in the extracellular space was then calculated from the extracellular space and the concentration of 2-DG in the media. The 2-DG in the extracellular space was subtracted from the true activity of ^3^H-labeled 2-DG in the skeletal muscle to calculate intracellular space. Glucose uptake was assessed by taking into account the concentration of unlabeled 2-DG in the media, the intracellular space, protein concentration of the skeletal muscle, and the incubation time.

### Statistical Analysis

Mice were randomized to treatment groups. The exercise trained mice were divided into treatment groups in a balanced manner based on their running distance to obtain equal running distances in each group. The experiments and subsequent analysis were all performed in a balanced but not blinded manner. Statistical analyses were performed using unpaired/paired Student’s *t*-tests or factorial (i.e., paired/unpaired 2 × 2 or 3 × 3) analysis of variance (ANOVA) as appropriate. When unequal variance was found between groups, the data was transformed to obtain equal variance. The Dunnett *post hoc* test was used for 1-way ANOVA whereas the Tukey *post hoc* test was used for 2-way and 3-way ANOVAs. *P* < 0.05 was considered statistically significant. Data are reported as mean ± standard error of mean (SEM).

## Results

### Circadian Fluctuations in Blood Glucose and Whole-Body Insulin Tolerance

**FIGURE 1:**
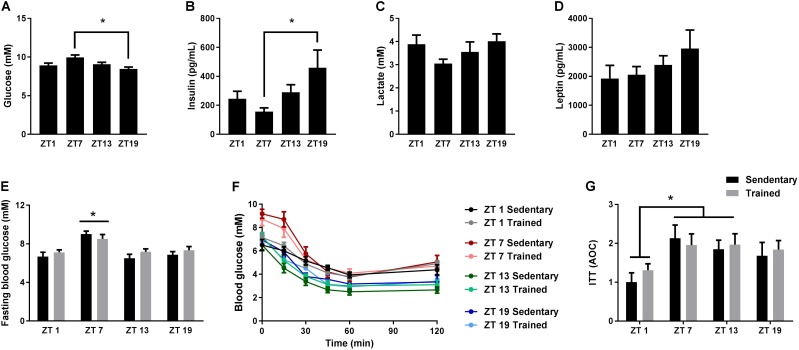
Circadian measurements of blood parameters and insulin tolerance. Mice were housed under standard conditions with a 12 h light/dark cycle and *ad libitum* access to food. Blood glucose **(A)**, insulin **(B)**, lactate **(C)**, and leptin **(D)** levels were measured on tail blood at zeitgeber time (ZT) 1, 7, 13, and 19. **(E)** Two hours fasting blood glucose measurements in sedentary mice and in mice which have had access to exercise training for 3 weeks (Trained). **(F)** Insulin tolerance test (ITT) on sedentary and exercise trained mice at ZT 1, 7, 13, and 19. **(G)** Area over the curve (AOC) for the ITT. Data shown is mean + SEM for *n* = 11–13 **(A–D)**, *n* = 8–9 **(E)**, and *n* = 6–8 **(F,G)**. ^∗^*p* < 0.05, 1-way ANOVA **(A–D)** and 2-way ANOVA with Tukey’s correction **(E,G)**.

Given the important role of circadian rhythms for metabolism, we examined the daily fluctuations of four different blood parameters over 24 h; glucose, insulin, lactate, and leptin. In undisturbed and unfasted mice, blood glucose levels were significantly increased at ZT 7 in the middle of the light phase (**Figure 1A**), whereas insulin levels were increased at ZT 19 in the middle of the dark phase (**Figure 1B**). Mice generally consume the bulk of their food during the first half of the dark phase (Sasaki et al., 2016), which is in accordance with the increased insulin levels at ZT 19. In the same mice, we did not observe significant differences in lactate or leptin levels at the four different time points (**Figures 1C,D**).

Next, we examined whether whole-body insulin tolerance followed a diurnal rhythm. Since exercise training influences insulin tolerance (Bradley et al., 2008), we also investigated the effect of exercise training on the daily pattern of insulin tolerance. Thus, we performed ITTs on cohorts of mice with or without access to running wheels for 3 weeks. Blood glucose levels after a 2 h fast were increased at ZT 7 compared to all other time points (**Figure 1E**). This increase was independent of training status. During the ITT, a smaller area over the curve (AOC) at ZT 1 was observed compared to ZT 7 and 13 (**Figures 1F,G**), indicating that insulin tolerance is reduced during the early light phase. Despite the fact that mice were running 4.3 ± 0.8 km on average per day, exercise training did not have a significant effect on insulin tolerance.

### Insulin and AMPK Signaling in Skeletal Muscle Follow a Diurnal Rhythm

**FIGURE 2:**
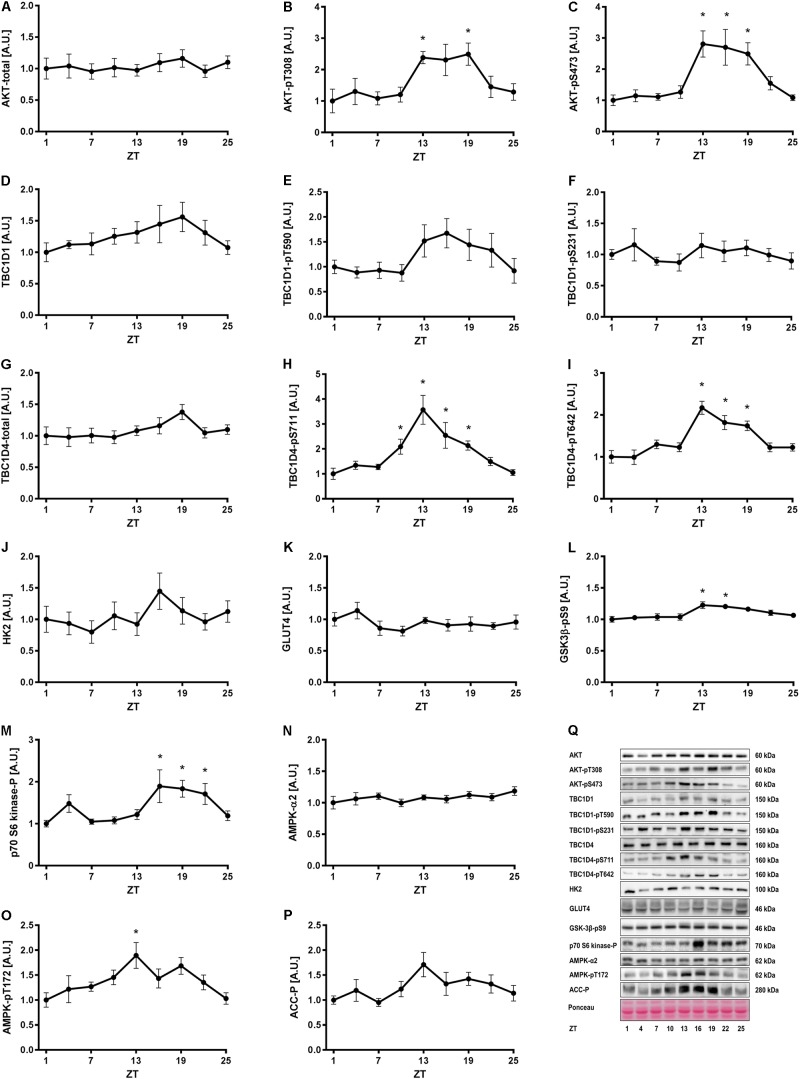
Insulin and AMPK signaling in skeletal muscle over 24 h. Mice had access to a running wheel for 3 weeks and were then sacrificed every third hour for 24 h for tissue collection. Activity of signaling pathways regulating insulin- and exercise-induced glucose uptake were analyzed in gastrocnemius muscle by measurement of total protein and the phosphorylation status of proteins involved in metabolic regulation. Protein levels of **(A)** total AKT, **(B)** AKT-pT308, **(C)** AKT-pS473, **(D)** total TBC1D1, **(E)** TBC1D1-pT590, **(F)** TBC1D1-pS231, **(G)** total TBC1D4, **(H)** TBC1D4-pS711, **(I)** TBC1D4-pT642, **(J)** HK2, **(K)** GLUT4, **(L)** GSK3β-pS9, **(M)** p70-S6 kinase-p, **(N)** total AMPK, **(O)** AMPK-pT172, and **(P)** ACC-p. **(Q)** Representative Western blots for data shown in **A–P**. Data shown are means + SEM for *n* = 6–8. ^∗^*p* < 0.05 from ZT 1, 1-way ANOVA with Dunnett’s correction.

To investigate whether insulin- and exercise-induced signaling pathways follow a diurnal rhythm in skeletal muscle, we studied gastrocnemius muscle of exercise trained mice (i.e., 3 weeks of voluntary exercise training in running wheels) every third hour for 24 h. Total AKT levels were unaltered over 24 h (**Figures 2A,Q**). However, AKT phosphorylation at S473 and T308 was increased at ZT 13–19 in skeletal muscle (**Figures 2B,C**). A similar phosphorylation pattern was observed for the AKT targets, TBC1 domain family member 1 (TBC1D1) and TBC1D4. The change in phosphorylation did not reach significance for TBC1D1 (**Figures 2E,F**), but TBC1D4 was significantly more phosphorylated on S711 and T642 at ZT 13, 16, and 19 compared to the beginning of the light phase (**Figures 2H,I**). Total TBC1D1 and TBC1D4 were unaltered over 24 h (**Figures 2D,G**). TBC1D1/4 are Rab-GTPase activating proteins, regulating translocation of glucose transporter 4 (GLUT4) to the plasma membrane (Sakamoto and Holman, 2008). The increase in TBC1D4 phosphorylation may indicate that glucose uptake is increased during the first half of the dark phase. Hexokinase 2 (*Hk2*) and *Glut4* expression is clock-dependent, with GLUT4 levels undergoing diurnal oscillations in skeletal muscle (Dyar et al., 2014; Harfmann et al., 2016). However, HK2 and GLUT4 protein levels did not oscillate over the time course studied (**Figures 2J,K**). Glycogen synthase kinase 3β (GSK3β), a direct target of AKT, showed a small but significant increase in phosphorylation on S9 at ZT 13 and 16 (**Figure 2L**). Thus, these data are consistent with the increased AKT phosphorylation in the early dark phase. p70 S6 kinase, which is phosphorylated by mammalian target of rapamycin complex 1 (mTORC1) downstream of AKT, showed increased phosphorylation levels at ZT 16–22 (**Figure 2M**). p70 S6 kinase forms a negative feedback loop that inhibits insulin signaling (Tremblay and Marette, 2001). The activation of p70 S6 kinase during the second half of the dark phase indicates insulin sensitivity may be decreased in this period.

Next, we investigated one of the signaling pathways induced by exercise. Total AMPK levels were stable over 24 h (**Figure 2N**), while AMPK phosphorylation oscillated significantly with increased phosphorylation at ZT 13 (**Figure 2O**). This was consistent with the increased phosphorylation of TBC1D4-S711 at ZT 10–19 (**Figure 2H**), since TBC1D4-S711 is a site phosphorylated by both AMPK and insulin (Treebak et al., 2010). However, phosphorylation of another AMPK target, acetyl-CoA carboxylase (ACC) (**Figure 2P**), and phosphorylation of TBC1D1 at the AMPK site S231 (**Figure 2F**), were unaltered over 24 h. This suggests AMPK trimer-specific diurnal activity patterns in skeletal muscle. Collectively, our results indicate that insulin- and exercise-induced signaling is augmented in the beginning of the dark period in mice with unlimited access to food and running wheels. The augmented signaling is likely due to increased food intake and activity in this period.

### *In vivo* Glucose Uptake of Skeletal Muscle and Adipose Tissue Follows a Different Diurnal Rhythm Compared to Whole-Body Insulin Tolerance

**FIGURE 3:**
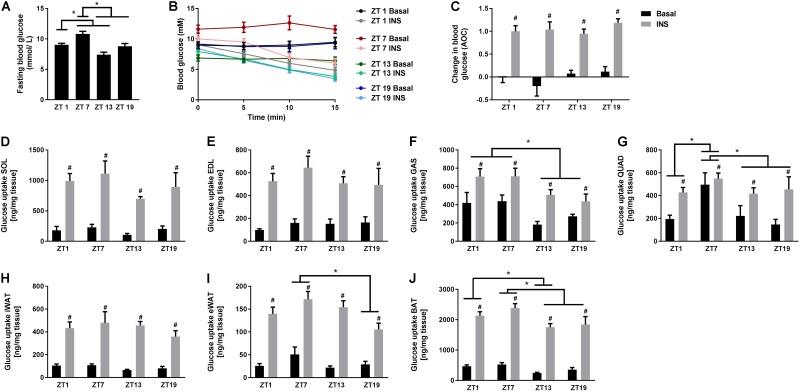
*In vivo* circadian measurements of basal and insulin-stimulated glucose uptake in skeletal muscle and adipose tissue. **(A)** Blood glucose measurements after a 2 h fast. **(B)** Change in blood glucose in response to saline or insulin (0.75 U/kg) at the four time points studied. **(C)** Area over the curve (AOC) for the data shown in **B**. Glucose uptake in skeletal muscle: **(D)** soleus (SOL), **(E)** extensor digitorum longus (EDL), **(F)** gastrocnemius (GAS), and **(G)** quadriceps (QUAD). Glucose uptake in adipose tissue: **(H)** inguinal white adipose tissue (iWAT), **(I)** epididymal WAT (eWAT), and **(J)** brown adipose tissue (BAT). Data shown are means + SEM for *n* = 6. ^∗^*p* < 0.05 between time points, ^#^*p* < 0.05 between basal and insulin-stimulated, 1-way ANOVA **(A)** and 2-way ANOVA with Tukey’s correction **(C–J)**.

To investigate whether the rhythmicity of insulin signaling and whole-body insulin tolerance was reflected in basal and insulin-stimulated glucose uptake in skeletal muscle and adipose tissue, we performed *in vivo* glucose uptake measurements. Similarly to the ITT results, we observed that fasting blood glucose levels were increased at ZT 7 (**Figure 3A**). However, in contrast to the ITT results, insulin stimulation reduced the blood glucose concentration to the same extent at all time points (**Figures 3B,C**). This inconsistency might be due to a difference in the time period measured, since blood glucose levels after 15 min in the ITT also did not differ significantly with circadian timing. Basal and insulin-stimulated glucose uptake was measured in four different skeletal muscles and three different adipose tissue depots (**Figures 3D–J**). We did not observe any oscillation in glucose uptake in either EDL or soleus muscle (**Figures 3D,E**), or inguinal white adipose tissue (iWAT) (**Figure 3H**). However, in gastrocnemius muscle, and independent of insulin stimulation, glucose uptake was significantly higher at two time points during the light phase (ZT 1 and 7) compared to the dark phase (**Figure 3F**). Moreover, in quadriceps muscle, glucose uptake was generally higher at ZT 7 compared to all other time points (**Figure 3G**), irrespectively of insulin stimulation, and in epididymal WAT (eWAT) glucose uptake was significantly higher at ZT 7 compared to ZT 19 (**Figure 3I**). In brown adipose tissue (BAT), glucose uptake was higher at ZT 1 compared to ZT 13, and at ZT 7 compared to ZT 13 and 19 (**Figure 3J**). Our finding that the insulin response did not differ between time points for any of the tissues indicates that insulin responsiveness to glucose uptake of the skeletal muscle and adipose tissue does not follow a diurnal pattern. However, basal and insulin-stimulated glucose uptake of several skeletal muscles and adipose tissues exhibited circadian rhythmicity with the highest uptake in the middle of the light phase.

### Skeletal Muscle Insulin Sensitivity and Signaling Is Increased During the Dark Phase

**FIGURE 4:**
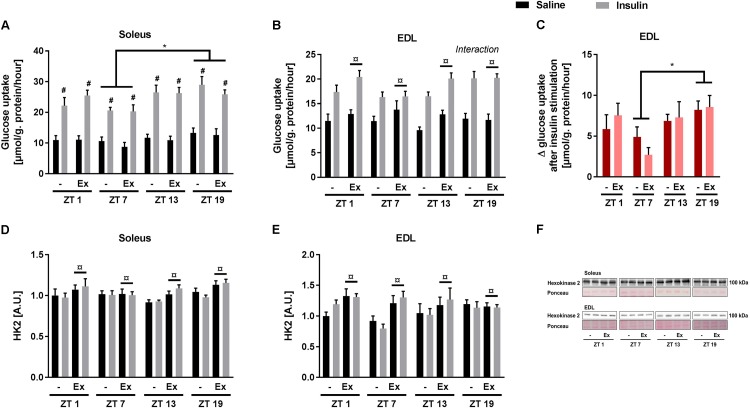
*Ex vivo* circadian measurements of insulin-stimulated glucose uptake and HK2 levels in skeletal muscle. Mice were either sedentary or had access to a running wheel for 4 weeks (Ex). Soleus and extensor digitorum longus (EDL) muscles were isolated at zeitgeber time (ZT) 1, 7, 13, and 19 and incubated under oxygenated conditions. Glucose uptake was measured under basal and submaximal insulin-stimulation (200 μU/mL) in **(A)** Soleus (3-way ANOVA; insulin-stimulation effect, *F* = 344, *p* < 0.0001, and time-of day effect *F* = 4.34, *p* = 0.008), and **(B)** EDL (3-way ANOVA; training effect, *F* = 5.31, *p* = 0.025, and time-of-day and insulin stimulation interaction, *F* = 3.644, *p* = 0.018). **(C)** Delta- (Δ) glucose uptake after insulin stimulation in EDL (insulin-stimulated glucose uptake subtracted basal glucose uptake). Protein levels of HK2 **(D)** in soleus (3-way ANOVA; insulin-stimulation effect, *F* = 9.78, *p* = 0.003) and **(E)** EDL (3-way ANOVA; insulin-stimulation effect, *F* = 8.04, *p* = 0.006). **(F)** Representative Western blots for HK2 in soleus and EDL. Data shown are means + SEM for *n* = 8. ^∗^*p* < 0.05 between time points, ^#^*p* < 0.05 between basal and insulin-stimulated, ^¤^*p* < 0.05 between sedentary and trained, 3-way ANOVA **(A,B,D,E)**, 2-way ANOVA with Tukey’s correction **(C)**.

Given the discrepancies between the diurnal pattern of insulin tolerance and skeletal muscle glucose uptake, we next studied the circadian rhythm of insulin sensitivity in an isolated system. Glucose uptake was measured *ex vivo* at the same four time points as in the *in vivo* experiments (i.e., ZT 1, 7, 13, and 19). In contrast to the *in vivo* studies, a submaximal insulin concentration was used to examine insulin sensitivity. Insulin augmented glucose uptake in both soleus and EDL muscle at all time points (**Figures 4A,B**). In soleus muscle, both basal and insulin-stimulated glucose uptake was higher at ZT 19 compared to ZT 7 (**Figure 4A**). In EDL muscle, there was an interaction between insulin stimulation and time-of-day (assessed by a repeated measures 3-way ANOVA), with this interaction being driven by an increased delta glucose uptake at ZT 19 compared to ZT 7 (**Figure 4C**). These results demonstrate a circadian pattern in skeletal muscle insulin sensitivity with increased insulin sensitivity at ZT 19. Wheel running increased overall glucose uptake in EDL muscle, but had no effect in soleus muscle. The circadian pattern of glucose uptake was not affected by exercise training in either of the two muscles. To confirm the effect of exercise training, protein levels of Hexokinase 2 (HK2) were measured in the incubated skeletal muscles (**Figures 4D–F**). HK2 levels are known to be increased in response to exercise training (Brandauer et al., 2013; Fentz et al., 2015). As expected wheel running increased HK2 levels in both soleus and EDL muscle.

**FIGURE 5:**
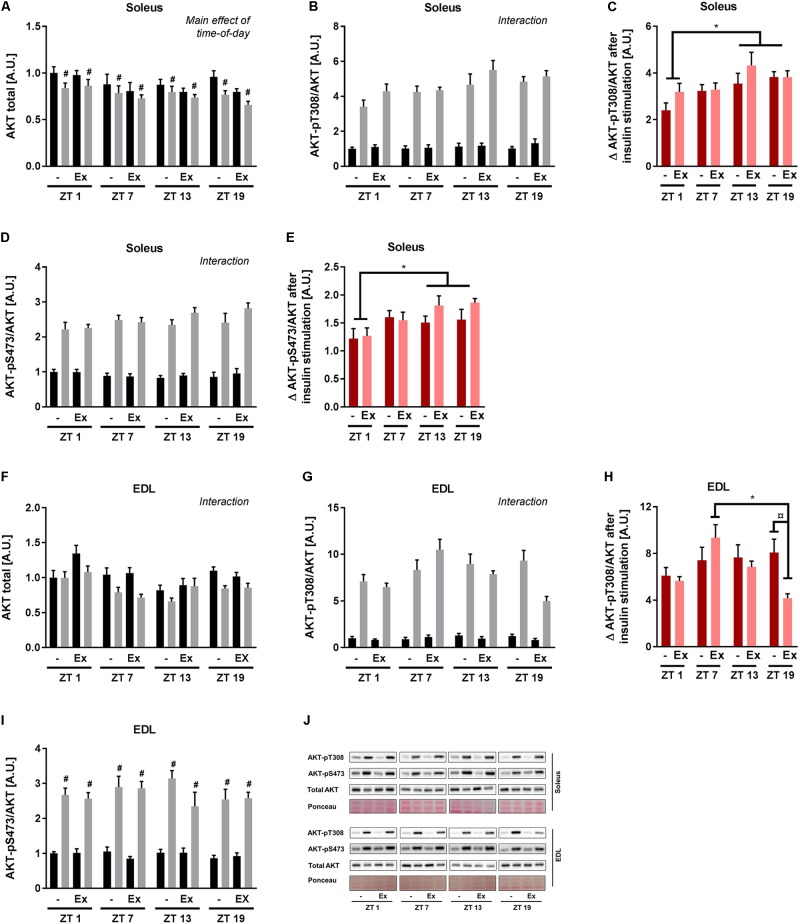
*Ex vivo* circadian measurements of insulin signaling in skeletal muscle. Mice were either sedentary or had access to a running wheel for 4 weeks (Ex). Soleus and extensor digitorum longus (EDL) muscles were isolated at zeitgeber time (ZT) 1, 7, 13, and 19 and incubated under basal and submaximal insulin-stimulation (200 μU/mL). Protein levels in soleus of **(A)** total AKT (3-way ANOVA; insulin-stimulation effect, *F* = 21.557, *p* < 0.0001, and time-of-day effect, *F* = 2.85, *p* = 0.046 with the *post hoc* test no specific difference was found with time-of-day), **(B)** AKT-pT308 normalized to total AKT (3-way ANOVA; time-of-day, and insulin stimulation interaction, *F* = 4.53, *p* = 0.007), and **(C)** phosphorylation on AKT-T308 after insulin stimulation subtracted unstimulated phosphorylation. Protein levels in soleus of **(D)** AKT-pS473 normalized to total AKT (3-way ANOVA; time-of-day and insulin stimulation interaction, *F* = 4.21, *p* = 0.009), and **(E)** phosphorylation on AKT-S473 after insulin stimulation subtracted unstimulated phosphorylation. Protein levels in EDL of **(F)** total AKT (3-way ANOVA; time-of-day, training and insulin-stimulation interaction, *F* = 2.81, *p* = 0.048), **(G)** AKT-pT308 normalized to total AKT (3-way ANOVA; time-of-day, training and insulin stimulation interaction, *F* = 3.55, *p* = 0.020), and **(H)** phosphorylation on AKT-T308 after insulin stimulation subtracted unstimulated phosphorylation. Protein levels in EDL of **(I)** AKT-pS473 normalized to total AKT (3-way ANOVA; insulin stimulation effect, *F* = 475, *p* < 0.0001). **(J)** Representative Western blots for data shown in **A–I**. Data shown are means + SEM for *n* = 8. ^∗^*p* < 0.05 between time points, ^#^*p* < 0.05 between basal and insulin-stimulated, ^¤^*p* < 0.05 between sedentary and trained, 3-way ANOVA **(A,B,D,F,G,I)**, 2-way ANOVA with Tukey’s correction **(C,E,H)**.

To clarify the signal transduction events associated with the observed circadian pattern of skeletal muscle insulin sensitivity, canonical insulin signaling was determined in the incubated soleus and EDL muscle. In soleus muscle, AKT protein levels differed with time-of-day and insulin stimulation (**Figure 5A**). To take these differences into account, phosphorylation of AKT was normalized to total AKT protein. Insulin stimulation induced AKT phosphorylation at T308 and S473 at all time points as expected (**Figures 5B,D**). A 3-way repeated measure ANOVA revealed interactions between time-of-day and insulin stimulation for both AKT phosphorylation sites. These interactions were driven by an increased delta phosphorylation at ZT 13 and 19 compared to ZT 1 (**Figures 5C,E**). In EDL muscle total AKT protein levels differed with time-of-day, training status, and insulin stimulation (**Figure 5F**). AKT-T308 phosphorylation levels showed an interaction between time-of-day, training status, and insulin stimulation (**Figure 5G**). This interaction was driven by an increased delta phosphorylation of AKT-T308 after insulin stimulation at ZT 7 compared to ZT 19 in the trained group, and an increased delta phosphorylation of AKT-T308 after insulin stimulation in the sedentary group compared to the trained group at ZT 19 (**Figure 5H**). In EDL muscle AKT-pS473 phosphorylation was induced by insulin stimulation, but there was no effect of time-of-day (**Figures 5I,J**). Our results indicate that exercise training induces circadian rhythmicity of insulin signaling in EDL muscle. However, this exercise training-induced rhythm does not follow the rhythm of insulin sensitivity in EDL muscle. Taken together, our results suggest that skeletal muscle cell-autonomous insulin sensitivity follows a circadian pattern with increased levels during the dark period.

## Discussion

Glucose handling and control differs throughout the day with enhanced glucose tolerance in the morning (Service et al., 1983; Lee et al., 1992; Saad et al., 2012). The deterioration of glucose tolerance during the day appears to result from both impaired insulin secretion and impaired insulin action later in the day (Service et al., 1983; Lee et al., 1992; Saad et al., 2012). However, it is not clear to what extent the diurnal fluctuations of glucose tolerance are reflected in changes in muscle insulin sensitivity. To determine whether skeletal muscle insulin sensitivity and signaling follow a circadian rhythm, diurnal responses were assessed in mice using both *in vivo* and *ex vivo* approaches. Our data validate that whole body insulin tolerance in mice follows a diurnal pattern with lowest response to insulin during the early light phase. Similar results have previously been reported (Shi et al., 2013; Zhou et al., 2014). Insulin signaling and sensitivity in isolated skeletal muscle peaked during the dark phase. These results indicate circadian rhythmicity of skeletal muscle insulin sensitivity.

Phosphoproteomic studies have established that a substantial percentage of phosphoproteins in metabolically active tissues, such as the liver, are regulated in a circadian manner (Robles et al., 2017). This circadian control of protein phosphorylation provides a mechanism by which tissues adapt to various environmental cues throughout the day. Here, we observed 24 h oscillations in the phosphorylation level of AKT and TBC1D4 in skeletal muscle *in vivo* with the highest response noted during the first 7 h of the dark phase. Similar diurnal oscillations in AKT-S473 phosphorylation have previously been reported (Dyar et al., 2014). AKT-S473 oscillation is unchanged in skeletal muscle-specific *Bmal1* knockout mice (Dyar et al., 2014), indicating that fluctuations in insulin signaling are mediated by hormonal changes, rather than directly by the clock machinery. We found that AMPK phosphorylation was also increased in the beginning of the dark phase, consistent with locomotor measurements indicating that mice are most active during this period (Sasaki et al., 2016). We detected increased p70 S6 kinase phosphorylation later during the night. p70 S6 kinase is a target of mTORC1. AKT signaling induces activation of mTORC1, whereas AMPK signaling inhibits its activation (Kimura et al., 2003). The increased phosphorylation of p70 S6 kinase therefore indicates that insulin signaling is dominating over AMPK signaling. A circadian phosphoproteome analysis of liver has revealed that phosphorylation and activation of AKT, mTORC1 and p70 S6 kinase is augmented in the dark phase (Robles et al., 2017). Together with our results, this indicates that the *in vivo* phosphorylation cycle of insulin signaling components follows a similar pattern in several tissues, which would be expected based on its dependency on the endogenous insulin levels. The diurnal rhythm of insulin signaling may affect the circadian clock in peripheral tissues. We observed diurnal oscillations in GSK3β phosphorylation in skeletal muscle. GSK3β has previously been shown to phosphorylate several core clock proteins including BMAL1, and hereby regulate their activity (Yin et al., 2006; Sahar et al., 2010; Besing et al., 2015).

The signaling pathways regulating the circadian changes in skeletal muscle insulin sensitivity are not clear, and neither is the connection to the core clock machinery. In isolated soleus muscle, basal and insulin-stimulated glucose uptake was decreased during the light phase, whereas insulin sensitivity was decreased in EDL muscle during the light phase. Decreased insulin-stimulated glucose uptake in isolated soleus muscle from *Bmal1* knockout mice has been ascribed to two pathways: (1) decreased expression of *Hk2* and *Tbc1d1* and hereby decreased protein levels of HK2, TBC1D1, and GLUT4 and (2) decreased expression of *Pdp1* and increased expression of *Pdk4*, which in turn would lead to decreased phosphorylation of the rate-limiting pyruvate dehydrogense (PDH) subunit PDH-Eα (Dyar et al., 2014). We and others did not observe significant circadian oscillations in HK2 and TBC1D1 levels (Dyar et al., 2014), which exclude the contribution of the first pathway as the explanation for the circadian oscillations in skeletal muscle insulin sensitivity. Regarding the second pathway, PDH activity or PDH-E1α phosphorylation does not show significant circadian oscillations in skeletal muscle (Dyar et al., 2014), rendering this pathway unlikely to be the main regulator. The changes in insulin sensitivity could also not be explained by changes in AKT signaling. In soleus muscle insulin-stimulated AKT phosphorylation at both T308 and S473 followed a similar pattern as the glucose uptake. However, since AKT phosphorylation in isolated EDL muscle did not follow the same rhythmicity as insulin sensitivity the mechanism is likely to be downstream of AKT. We hypothesize that this could be through AMPK-induced changes in TBC1D4 phosphorylation, since phosphorylation of the AMPK site S711 positively correlates with insulin sensitivity (Kjøbsted et al., 2015, 2017). This hypothesis is supported by our discovery of increased phosphorylation of TBC1D4-S711 in skeletal muscle during the dark phase *in vivo*. Based on our experiments, we cannot conclude to what extent the circadian changes in skeletal muscle insulin sensitivity is dependent on the clock machinery or by signaling pathways induced *in vivo*, e.g., by changes in physical activity level.

The importance of peripheral tissue insulin sensitivity for the circadian fluctuations in whole-body insulin tolerance is not clear. We observed a different diurnal pattern of insulin-stimulated glucose uptake in skeletal muscle *in vivo* compared to whole-body insulin tolerance. Furthermore, whole-body insulin tolerance was high when skeletal muscle insulin sensitivity to glucose uptake *ex vivo* was low. This indicates that even though there are circadian changes in insulin sensitivity, skeletal muscle is probably not the primary organ responsible for the circadian changes in whole-body insulin tolerance. Human adipose tissue explants show circadian rhythmicity in insulin-stimulated AKT phosphorylation, with the highest sensitivity observed in the middle of the day; corresponding to the time-of-day where insulin tolerance is also high (Carrasco-Benso et al., 2016). This finding highlights adipose tissue as a candidate organ responsible for the circadian changes in insulin tolerance. However, we did not observe an increase in glucose uptake in adipose tissues at ZT 13 when insulin tolerance is high, which makes it less likely that adipose tissue is responsible for the circadian changes in whole-body insulin tolerance. Another candidate tissue which could be responsible for the diurnal pattern of insulin tolerance is the liver. The importance of the liver for the circadian changes in insulin tolerance has been demonstrated by use of liver-specific *Bmal1* knockout mice. These mice have reduced insulin tolerance and lost circadian rhythmicity in insulin tolerance (Lamia et al., 2008; Zhou et al., 2014). Circadian changes in liver insulin sensitivity can partly explain the discrepancy between whole-body glucose disposal during the ITT and the insulin-stimulated glucose uptake measured *in vivo* in skeletal muscle and adipose tissue. Enhanced liver insulin sensitivity during the dark phase will lower endogenous glucose production (EGP), which may counter-act the decreased glucose uptake in the peripheral tissues to some extent. Decreased EGP during the active dark period has been described (Coomans et al., 2013). However, decreased EGP is not the sole explanation, since glucose disposal is increased in the middle of the dark period, suggesting a role for peripheral tissues (Coomans et al., 2013). Collectively, our results indicate that neither skeletal muscle nor adipose tissue play a major role for the circadian rhythmicity in whole-body insulin tolerance.

We hypothesized that exercise training would potentiate the circadian rhythm of insulin sensitivity in skeletal muscle. This hypothesis was based on the emerging evidence of a circadian phase shift in response to exercise (Wolff and Esser, 2012; Yasumoto et al., 2015). However, we did not observe an effect of voluntary exercise training on the circadian rhythm of insulin tolerance or insulin sensitivity of incubated soleus and EDL muscles. In general, exercise training appeared to have little effect on insulin sensitivity under the experimental conditions used, which might explain its lack of effect on the rhythmicity. Exercise training, however, increased HK2 levels in soleus and EDL muscle and enhanced glucose disposal independent of insulin in EDL muscle. Moreover, exercise training induced a circadian rhythmicity of insulin signaling in incubated EDL muscle with an increased AKT-T308 phosphorylation at ZT 7 compared to ZT 19. This difference is most likely driven by the reduced response to insulin in the trained group at ZT 19. Neither AKT-S473 phosphorylation nor glucose uptake was affected by the decreased AKT-T308 phosphorylation in the trained group. However, the contribution to other pathways cannot be excluded. As we only studied voluntary exercise training, a condition where mice primarily run in the first half of the dark period (Sasaki et al., 2016), it would be interesting to determine the effect of exercise training performed at other time intervals of the day. Evidence is emerging that exercise at the right time of the day can correct “misalignment” of the clock (Mrosovsky and Salmon, 1987; Schroeder et al., 2012). Thus, forced activity at time intervals where the mice are not normally active may have larger effects on circadian rhythms.

## Conclusion

We report distinct circadian rhythmicity between whole body insulin tolerance and *ex vivo* skeletal muscle insulin sensitivity to glucose transport and signaling. Insulin tolerance was increased in the middle of the light phase and in the early dark phase, whereas insulin sensitivity was highest during the dark phase. Furthermore, our results indicate that neither skeletal muscle nor adipose tissue play a major role for the circadian rhythmicity in insulin tolerance, since *in vivo* glucose uptake of skeletal muscle and adipose tissue follows a different diurnal rhythm compared to whole-body insulin tolerance. Finally, we have demonstrated that both the diurnal rhythm of whole-body insulin tolerance and skeletal muscle-specific insulin sensitivity is independent of voluntary exercise training.

## Author Contributions

AB and JTT conceived and designed the experiments. AB, ED, and LL performed the experiments. AB, ED, ZG-H, JZ, and JTT analyzed and interpreted the data. AB prepared the figures and drafted the manuscript. All authors read, revised, and approved the final manuscript.

## Conflict of Interest Statement

The authors declare that the research was conducted in the absence of any commercial or financial relationships that could be construed as a potential conflict of interest.
